# Characterizing microbiota and metabolomics analysis to identify candidate biomarkers in lung cancer

**DOI:** 10.3389/fonc.2022.1058436

**Published:** 2022-11-15

**Authors:** Bo Liu, Yige Li, Lijun Suo, Wei Zhang, Hongyun Cao, Ruicai Wang, Jiahui Luan, Xiaofeng Yu, Liang Dong, Wenjing Wang, Shiyang Xu, Shiyong Lu, Mei Shi

**Affiliations:** ^1^ State Key Laboratory of Microbial Technology, Shandong University, Qingdao, China; ^2^ Department of Pulmonary and Critical Care Medicine, Department of Clinical Microbiology, Zibo City Key Laboratory of Respiratory Infection and Clinical Microbiology, Zibo City Engineering Technology Research Center of Etiology Molecular Diagnosis, Zibo Municipal Hospital, Zibo, China; ^3^ Shandong University-Zibo Municipal Hospital Research Center of Human Microbiome and Health, Zibo, China; ^4^ Department of Pulmonary and Critical Care Medicine, Shandong Institute of Respiratory Diseases, The First Affiliated Hospital of Shandong First Medical University, Shandong Provincial Qianfoshan Hospital, Shandong University, Jinan, China; ^5^ Department of Thoracic Surgery, Zibo Municipal Hospital, Zibo, China; ^6^ Department of Pathology, Zibo Municipal Hospital, Zibo, China

**Keywords:** lung cancer, lung microbiota, 16S rRNA amplicon sequencing, metabolomics, biomarker

## Abstract

**Background:**

Lung cancer is the leading malignant disease and cause of cancer-related death worldwide. Most patients with lung cancer had insignificant early symptoms so that most of them were diagnosed at an advanced stage. In addition to factors such as smoking, pollution, lung microbiome and its metabolites play vital roles in the development of lung cancer. However, the interaction between lung microbiota and carcinogenesis is lack of systematically characterized and controversial. Therefore, the purpose of this study was to excavate the features of the lung microbiota and metabolites in patients and verify potential biomarkers for lung cancer diagnosis.

**Methods:**

Lung tissue flushing solutions and bronchoalveolar lavage fluid samples came from patients with lung cancer and non-lung cancer. The composition and variations of the microbiota and metabolites in samples were explored using muti-omics technologies including 16S rRNA amplicon sequencing, metagenomics and metabolomics.

**Results:**

The metabolomics analysis indicated that 40 different metabolites, such as 9,10-DHOME, sphingosine, and cysteinyl-valine, were statistically significant between two groups (VIP > 1 and *P* < 0.05). These metabolites were significantly enriched into 11 signal pathways including sphingolipid, autophagy and apoptosis signaling pathway (*P* < 0.05). The analysis of lung microbiota showed that significant changes reflected the decrease of microbial diversity, changes of distribution of microbial taxa, and variability of the correlation networks of lung microbiota in lung cancer patients. In particular, we found that oral commensal microbiota and multiple probiotics might be connected with the occurrence and progression of lung cancer. Moreover, our study found 3 metabolites and 9 species with significantly differences, which might be regarded as the potential clinical diagnostic markers associated with lung cancer.

**Conclusions:**

Lung microbiota and metabolites might play important roles in the pathogenesis of lung cancer, and the altered metabolites and microbiota might have the potential to be clinical diagnostic markers and therapeutic targets associated with lung cancer.

## Introduction

Lung cancer (LC) is the main cause of death from cancer worldwide, and its incidence has continued to rise in recent years. Each year, more than 2.2 million people are diagnosed with the disease and 1.79 million die from it (1). The incidence is still rising in China, compared with declines in some western countries, which constitutes a major public health problem and causes a huge social burden (2). In contrast to small cell lung cancer (SCLC), which has been declining in many countries, non-small cell lung cancer (NSCLC) has accounted for the largest proportion of LC (80%-90%) currently (3). For now, common diagnostic methods contain x-ray film, positron emission computed tomography (PET), computed tomography (CT), and computer aided detection and diagnosis (CAD) (4). However, these common techniques, which are conveniently used by medical staff, lack specificity and accuracy. Biomarkers were found to have the potential to assist in the early diagnosis of LC. The most widely used and reliable biomarkers are protein biomarkers found in blood and bronchoalveolar lavage fluid (5). Combining biomarkers, imaging omics and artificial intelligence to constitute an integrated model for LC screening and diagnosis might be the progression orientation for ameliorating LC prediction in the future.

The crucial risk factors of LC contain tobacco smoking, environmental and occupational pollution exposure, chronic lung disease, and lifestyle factors (6). Emerging studies have indicated the lung microbiota and metabolites could affect pulmonary health and diseases of the lungs. Lungs were considered as a sterile environment for a long time due to the limit of the culture-based techniques. However, the use of 16S ribosomal RNA (rRNA) amplicon sequencing has led to the increase of the interest in the lung microbiota (7). Numerous studies have shown that the lung microbiota might play a crucial part in the pathogenesis of pulmonary diseases. Liu et al. showed that the lung microbial composition and community structures of smokers with LC were distinct from that of emphysema-only patients: the abundance of Proteobacteria in the lungs of patients with LC was significantly lower and the abundances of *Streptococcus* and *Prevotella* were higher compared to patients with emphysema only (8). Tsay et al. found that *Streptococcus* and *Veillonella* were up-regulated in the lower airways of LC patients, which was related to the promotion of ERK and PI3K signal pathways (9). Moreover, studies had demonstrated a lower alpha diversity of lung microbiota in LC patients compared with that in patients with non-lung cancer (10). In general, compared with the studies on the gut, there were fewer studies on the correlation between lung microbiota and pulmonary homeostasis and diseases.

Recently, the relationships between the progression of chronic inflammatory diseases and the variations of microbiota have been gradually discovered, and the lung diseases involved include cystic fibrosis, asthma and chronic obstructive pulmonary disease (COPD) (11). The relationship between smoking, airflow obstruction, and LC was well recognized. Previously study showed that COPD was an important element in LC risk in smokers and smokers with COPD had a 3- to 10-fold increased risk of developing LC compared to smokers without emphysema or significant airway obstruction (12). Prolonged exposure to environmental pollutants could stimulate inflammatory factors, promote the formation of the environment suitable for the survival of pathogenic bacteria, and lead to the dysbiosis of lung microbiota; the dysbiosis could further induce inflammation and tissue damage, ultimately leading to accelerated decline in lung function (13). However, current researches had focused on the association of the lung microbiota with chronic inflammatory disease of the lungs, there have been fewer studies on LC.

In recent years, several studies have generated interest in the relationships between metabolites of the lung microbiota and lung health. Microbial components can contribute to the progression of the pulmonary diseases by producing metabolites with oncogenic potential. Gao et al. showed that the metabolites produced by *Pseudomonas aeruginosa* might be related to the pathogenesis of cystic fibrosis (14). In addition, the lung microbiota and metabolites contribute to the maintenance of the balance of the host lung immune system, which is an important contributor to defend against infection. Steed et al. found that desaminotyrosine (DAT), which was a metabolite associated with microbiota, helped the host defend against influenza by positively stimulating type I IFN (15). However, the current LC related metabolomics studies mostly targeted metabolites such as plasma proteins, which might not characterize the metabolism of lung microenvironment clearly.

Despite recent emerging studies on the correlation between lung microbiota and metabolites associated with LC, the mechanisms still need to be further clarified. In addition, few studies have considered both lung microbiota and metabolites to explore their possible associations and their roles in the pathogenesis of LC. Therefore, in our study, the differences in lung microbiota and metabolites between LC patients and patients with non-lung cancer were explored by 16S rRNA amplicon sequencing and metagenomics. Moreover, we used the samples of lung tissue flushing solutions, which could characterize the metabolic changes of lung microenvironment more clearly, for the analysis of metabolomics to explore their effects on the development of LC. The results indicated that lung microbiota and metabolites might play key roles in the development of LC, and the altered metabolites and microbiota might have the potential to be clinical diagnostic markers and therapeutic targets associated with LC.

## Materials and methods

### Participants

From 2020 to 2021, patients with LC were recruited in the Zibo Municipal Hospital. The exclusion criteria included the uses of antibiotics, corticoids, probiotics, prebiotics or immunosuppressive drugs in the past 3 months; hypertension; diabetes; previous airway surgery; preoperative radiotherapy and chemotherapy; and atomization treatment. Non-lung cancer patients were set as the control group of the metabolomics, and the exclusion criteria were the same as those in the LC group. This study was approved by Ethics Committee of Zibo Municipal Hospital (No. 20201102), and each subject signed a voluntary informed consent before the study. The clinical information was summarized in **Supplementary Tables S1–S3**.

### Sample collection

Nine LC patients with unilateral tumors were selected from patients examined by bronchoscopy for the tests of 16S rRNA amplicon sequencing and metagenomics. All patients underwent routine examinations before operation, including electrocardiogram, pulmonary function, blood routine. Sterile saline samples of bilateral lungs were obtained by bronchoscopy in patients with LC. Paired samples of bronchoalveolar lavage fluid (BALF) included the one from the cancerous lobe and the other from the contralateral noncancerous lobe. Thirty LC patients with lung tumors and thirteen patients with non-lung cancer who underwent lobectomy were selected for the test of metabolomics. A whole tumor of 1 cm^3^ and healthy tissue located 5 cm from tumor in the same pulmonary region were extracted for each patient. The removed tumors and tissues were immediately flushed with sterile normal saline and collected in sampling tubes.

All samples were immediately stored at -80 °C until DNA extraction was performed.

### Non-targeted metabolomics profiling

Metabolites were extracted from the lung tissue flushing solutions and tested with a liquid chromatography-tandem mass spectrometry (LC-MS/MS). The metabolomics analysis was performed by UHPLC -Q Exactive HF-X system with a ACQUITY UPLC HSS T3 column (Waters, Milford, USA). The temperature of the column was set to 40°C and the injection volume was 2L. The flow rate of helium carrier gas was 0.4 mL/min, and the MS scanning range was m/z 70 - 1050. Progenesis QI (Waters Corporation, Milford, USA) was used to preprocess the MS raw data, and the obtained data matrix included retention time (RT), mass/charge ratio (M/Z) and peak intensity.

Principal component analysis (PCA) and orthogonal partial least squares-discriminant analysis (OPLS-DA) were used to explore whether all samples can be significantly clustered in different groups. The variable importance in projection (VIP) values of OPLS-DA and the P-values (Wilcoxon rank-sum test) were calculated to check the metabolites with statistically significant differences between two groups (16). Metabolites with P-values below 0.05 and VIP values above 1.00 were identified as differentially expressed metabolites. Metabolic pathway analysis was carried out to recognize the enriched pathways based on the altered metabolites. Altered metabolites were annotated through the KEGG database (https://www.kegg.jp/kegg/pathway.html) and the Python package SciPy was used for the pathway enrichment analysis. P-value were corrected by false discovery rate (FDR) with FDR ≤ 0.01 as the threshold.

### 16S rRNA amplicon sequencing

Microbial DNA was obtained from BALF samples using the FastDNA Spin Kit (MP Biomedicals, Shanghai, China) and tested for DNA purity using Nanodrop microspectrophotometer (Nanodrop 2000, Thermo Fisher Scientific, America). Finally, DNA integrity was determined using agarose gel electrophoresis. PCR was performed to amplification of the V3-V4 hypervariable regions of the bacterial 16S rDNA gene according to universal primers (338F: 5’- ACTCCTACGGGAGGCAGCAG- 3’, 806R: 5’- GGACTACHVGGGTWTCTAAT- 3’) that contained barcode (data available at Sequence Read Archive: PRJNA858534). PCR products were purified using the AxyPrep DNA Glue recovery kit, and the quantification and qualification of PCR products were detected on 2% agarose gels. Miseq libraries were constructed using NEXTFLEX DNA rapid Sequencing Kit and sequenced using Illumina’s Miseq PE300 high-throughput sequencing platform. Raw data were demultiplexed, quality-filtered by fastp (https://github.com/OpenGene/fastp), and merged using FLASH (http://www.cbcb.umd.edu/software/flash). UPARSE (version 7.1 http://drive5.com/uparse/) software was used to perform OTU clustering on all sequences with 97% similarity as standard.

The three diversity indices (Shannon, Chao, ace) of the samples were calculated and averaged to assess the level of alpha diversity in different groups which were obtained by Mothur and visualized by R. The β-diversity was analyzed by weighted UniFrac phylogenetic distance matrices, visualized in non-metric multidimensional scaling analysis (NMDS) plots and determined by Partial Least Squares Discriminant Analysis (PLS-DA) for statistical significance. The effect of the abundance of the species on the discrepancy between groups was estimated using linear discriminant analysis (LDA) and formed a table (LDA 2.0, *P* < 0.05). Wilcoxon rank-sum test was carried out to compare species differences between groups (*P* < 0.05).

Correlation networks were used to show changes in interactions between microbial communities. Degree (DC), closeness (CC), and betweenness centrality (BC) were used to describe the characteristics of multiple networks.

### Metagenomics analysis

Microbial DNA was obtained from BALF samples using the FastDNA Spin Kit (MP Biomedicals, Shanghai, China) and tested for DNA purity using Nanodrop microspectrophotometer (Nanodrop 2000, Thermo Fisher Scientific, America). Finally, DNA integrity was determined using agarose gel electrophoresis. DNA was fragmented to an average size of approximately 400 bp using Covaris M220 (Gene Company Limited, China) for paired-end library construction. DNA libraries were subsequently constructed and assessed using the NEXTFLEX Rapid DNA-Seq kit (Bioo Scientific, USA). The metagenomics sequencing was carried out on Illumina NovaSeq/Hiseq Xten (Illumina, USA, data available at Sequence Read Archive: PRJNA858501). The raw sequence reads were trimmed, and the clean reads were assembled *via* MEGAHIT. Gene prediction was performed using MetaGene (http://metagene.cb.k.u-tokyo.ac.jp/), and CD-HIT software (version 4.6.1 http://www.bioinformatics.org/cd-hit/) was used for predicting gene sequence clustering. Redundant gene sets were constructed using the longest sequence of each group clustered in DNA as representative. DIAMOND (https://github.com/bbuchfink/diamond) was employed to compare the sequences of non-redundant gene sets with Eggnog database (http://eggnog.embl.de/) to obtain the Clusters of Orthologous Groups (COG) functions corresponding to genes, and the relative abundance of the COG was calculated using the sum of gene abundances corresponding to COG.

Linear regression analysis was carried out to estimate the consistency between species and function. Significantly differences of COG categories between groups were detected by Wilcoxon rank-sum test (*P* < 0.01).

### Biomarker identification

Biomarker identification was performed by MetaboAnalyst (https://www.metaboanalyst.ca/) (17). Based on the differential metabolites and microbiota obtained by the above analysis, the Receiver Operating Characteristic curve (ROC) analysis was used to obtain curve and calculate the area under the curve (AUC). In addition, we combined the obtained biomarkers to further explore the predictive ability of the model.

## Results

### Metabolomics profiles change in LC patients

**Figure 1:**
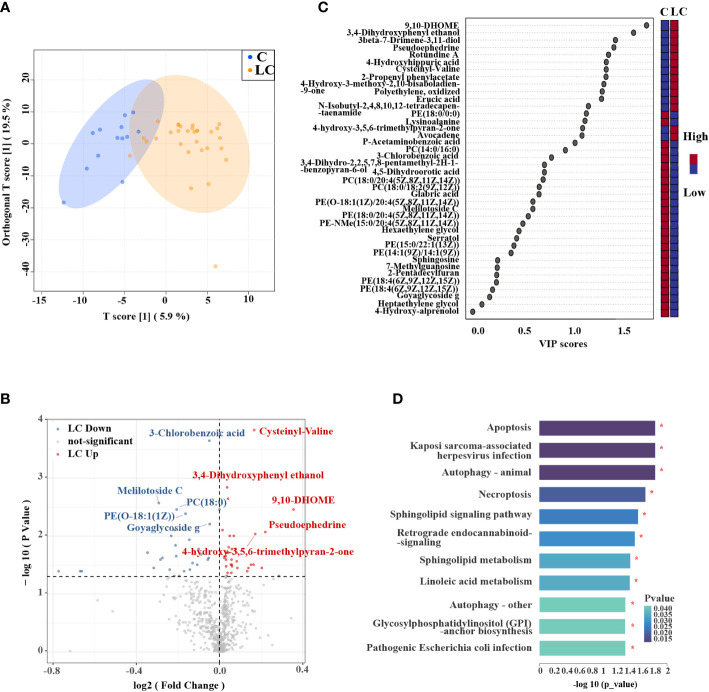
Metabolic profiles in LC patients and controls. **(A)** OPLS-DA showed that LC patients and controls were separated into two distinct clusters. **(B)** Volcano plot of metabolites of LC patients compared to controls. The y-axis represented p-value converted to negative log 10 scale and the x-axis represented log2 fold change. **(C)** Variable Importance in Projection (VIP) plot generated from the OPLS-DA analysis showed the most discriminative metabolites in descending order of importance. **(D)** KEGG pathway enrichment analysis of significantly different metabolites showed that there were 11 pathways had significant changes.

The samples of lung tissue flushing solutions were used for the analysis of metabolomics. Based on the processing of the raw data, the area under the curve was used to quantify peaks. In positive (ESI+) modes, 8,650 positive peaks were detected, 428 metabolites were identified, and 125 metabolites were annotated compared with KEGG database. In negative (ESI-) modes, 5,580 negative peaks were detected, 178 metabolites were identified, and 55 metabolites were annotated compared with KEGG database. The data was normalized and relative standard deviation (RSD) was used to evaluate the exclusion of data with poor stability during the experiment. Results indicated that favorable stability was tested from samples in the positive and negative modes **(** **Supplementary Figures S1A, B** **)**. PCA analysis revealed that QC samples were clearly differentiated, indicating the metabolomics datasets had satisfying stability and repeatability **(** **Supplementary Figures S1C, D** **)**. OPLS-DA analysis showed that the separation of metabolites in the samples of the two groups was obvious **(** **Figure 1A** **)**. Metabolite features that distinguished LC patients from controls were selected based on a log2 fold change cutoff at 1, and VIP scores determined by OPLS-DA **(**VIP > 1, *P* < 0.05, **Supplementary Table S4**). We obtained 40 metabolites with significant differences in relative abundance between LC patients and controls **(** **Figures 1B, C** **)**, which included 4 organic oxygen compounds, 4 fatty acyls, 3 organoheterocyclic compounds, 3 prenol lipids, 10 glycerophospholipids, 4 benzene and substituted derivatives, 2 carboxylic acids and derivatives, 1 benzenoid, 2 lipids and lipid-like molecules, 2 organic acids and derivatives, 1 purine nucleoside, and 4 other compounds **(** **Supplementary Figure S1E** **)**. Overall, 14 and 26 metabolites were significantly up-regulated and down-regulated in LC patients, respectively. Several fatty acyls such as 9,10-DHOME, Erucic acid and N-Isobutyl-2,4,8,10,12-tetradecapentaenamide presented at higher levels in LC patients, and some glycerophospholipids such as PC (14:0/16:0) and PE (14:1(9Z)/14:1(9Z)) were down-regulated in LC patients.

KEGG pathway enrichment analysis was performed to explore the metabolic pathways associated with differential metabolites in LC patients and controls. 130 metabolic pathways were identified, among which 24 metabolic pathways had significant differences, including ABC transporters, protein digestion and absorption, central carbon metabolism in cancer (*P* < 0.01, **Supplementary Figure S1F**). The significantly different metabolites were enriched into a total of 15 signal pathways, of which 11 signaling pathways were observably changed in LC patients, comprising autophagy, apoptosis, necroptosis and sphingolipid signaling pathway (*P* < 0.05, **Figure 1D**).

### Altered composition of the lung microbiota in LC patients

BALF samples were used for 16S rRNA amplicon sequencing to explore the changes of lung microbiota in LC patients and a total of 16 samples passed quality control and were included in the study. According to Usearch statistics, in the raw data of 16S rDNA sequencing using primers 338F and 806R, the total reading of each sample was 888,409 pairs. The original data were filtered by QIIME software and then spliced by FLASH software to generate tags sequence. A total of 16 qualified samples were obtained by BALF sample sequencing, with an average length of 425 bases. Finally, Uparse software was used to cluster the spliced sequences into OTUs according to 97% similarity, and the total number of OTUs obtained was 1,711.

**Figure 2:**
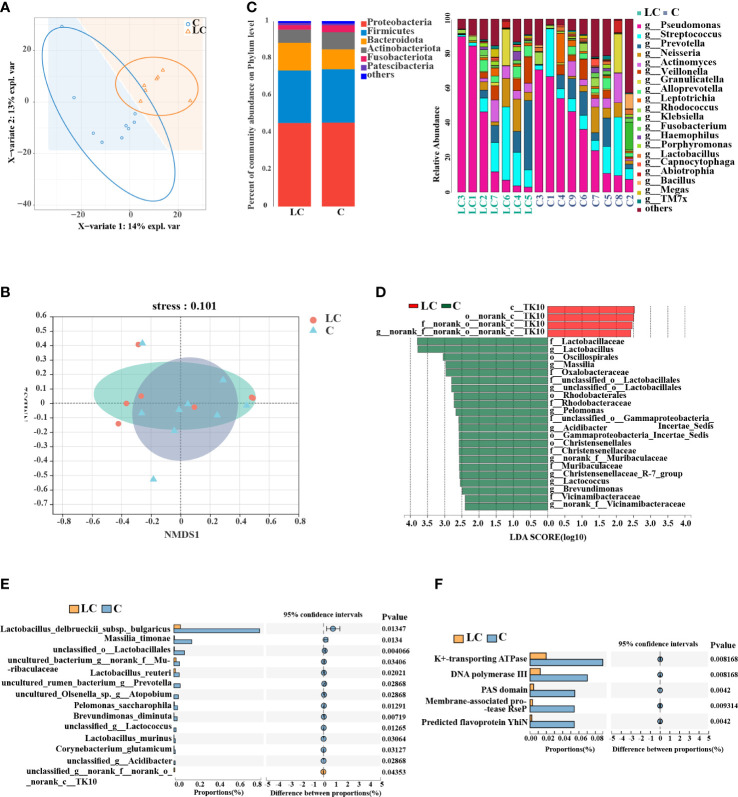
Lung microbiota composition profiles in LC patients and controls. **(A)** PLS-DA score plot of LC patients and controls showed clear distinction. **(B)** Non-metric multidimensional scaling analysis (NMDS) of the weighted UniFrac phylogenetic distance matrices demonstrated that the LC patients and controls showed two distinct clusters. **(C)** Relative abundance of major phyla and genera across BALF samples. **(D)** Differential taxa at the genus level identified by linear discriminant analysis (LDA) effect size (LEfSe) analysis (LDA > 2.0, *P* < 0.05). **(E)** Differential taxa at the species level identified by the Wilcoxon rank test (*P* < 0.05). **(F)** COG functional analysis of the microbiota between LC patients and controls.

Species cumulative curve and rarefaction curve at the OTU level indicated that the vast majority of microbial diversity was obtained in all samples **(** **Supplementary Figures S2A, B** **)**. Venn diagram was used to show the variation in OTUs between the two groups **(** **Supplementary Figure S2C** **)**. Overall, 453 OTUs were shared between groups and there were more unique OTUs in controls (973) than in the LC patients (285). The results of PLS-DA model analysis reflecting the clustering of the two groups showed that the separation between LC patients and controls was obvious **(** **Figure 2A** **)**. The alpha diversities of two groups did not show a significant difference **(** **Supplementary Figure S2D** **)**. NMDS analysis on the basis of Bray-Curtis similarity distance indicated that the two groups were apart from each other on the ordination (stress<0.2, **Figure 2B**). A taxonomic analysis of sequences revealed that the most prevalent phylum in the lung microbial community was Proteobacteria and variations of microbial composition at the genus level between individuals could be seen **(** **Figure 2C**, **Supplementary Table S5** **)**.

We relied on LEfSe analysis to identify the major taxa that influenced the differences between the two groups and two-sided Welch’s t-test. LEfSe analysis recognized 26 genera which had discrepant abundances between the two groups (LDA > 2.0, *P* < 0.05). In LC patients, an enrichment in Chloroflexi taxa was observed and *Lactobacillus, Massilia, Lactococcus*, Oscillospirales, Christensenellaceae were significantly more abundant in controls **(** **Figure 2D** **)**. Additionally, the results of the two-sided Welch’s t-test showed that *Lactobacillus delbrueckii subsp. bulgaricus, Massilia timonae, Lactobacillus reuteri* were more abundant in controls by species taxa (*P* < 0.05, **Figure 2E**).

Taken together, we identified some microbiota and metabolites that were different between two groups and their changes may be correlated. Therefore, a heat map showed the association between 20 differential genera and 40 differential metabolites closely related with the progression of LC (**Supplementary Figure S2E**).

Then, we used metagenomics analysis to predict gene functions of the lung microbiota and a total of 12 samples met the criteria after quality inspection. Based on the construction of non-redundant gene sets, we obtained 12, 671 genes with a total sequence length of 6, 216,664 (bp) and an average sequence length of 490.62. 179 different COG functional categories were identified (*P* < 0.05, **Supplementary Table S6**), and there were 5 functional categories had significant differences (*P* < 0.01, **Figure 2F**), including K^+^-transporting ATPase, DNA polymerase III, PAS domain, membrane-associated protease RseP and predicted flavoprotein YhiN. Linear regression analysis of the relationship between the similarity in the functional attributes of the community and community composition indicated that there is a prominent correlation between the two parts (R^2^ > 0.8, *P* < 0.01, **Supplementary Figure S2F**).

### Microbial interaction networks in non-lung cancer and lung cancer patients

**Figure 3:**
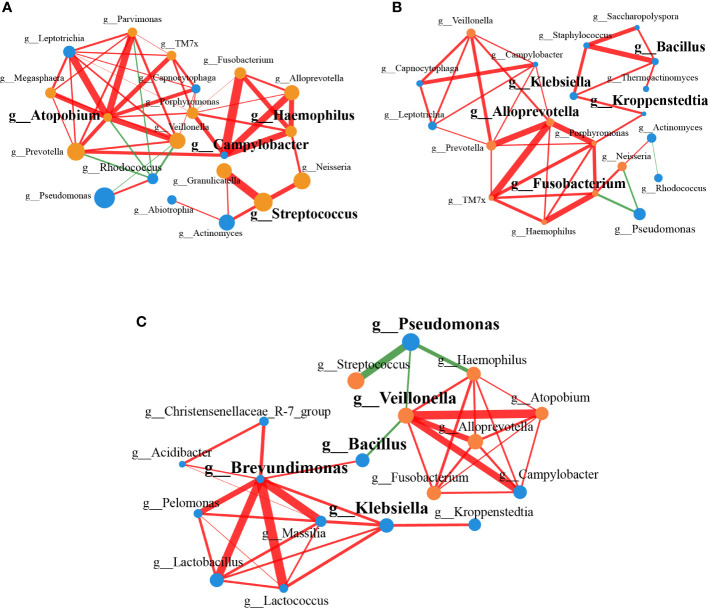
Correlation networks of genus associated with LC. **(A)** The correlation network between genera in LC patients (n=20). **(B)** The correlation network between genera in controls (n=20). **(C)** The correlation network based on the significantly different genera found by 16S rRNA amplicon sequencing (n=18, *P* < 0.05). The color of the nodes represented different groups, with nodes of the same color belonging to the same group. The yellow nodes represented LC group, and the blue nodes represented controls. The size of the nodes indicated the mean abundance of the genera in samples within the group. Edges between nodes represented the correlation between the abundance of two genera. Red lines represented positive correlations, while green lines represented negative correlations. The widths of the edges were proportional to the correlation strength, and wide line indicated stronger correlation.

To identify the interactions of the lung microbiota in patients with or without lung cancer, we constructed the correlation networks of genus taxa. The networks showed different bacterial interactions in the two groups, especially the network of LC patients was more complex than that of the controls. Given the distinct microbial composition between two groups, we compared the topology of the networks in each group. The number of mean degree and transitivity were higher in the LC patients (mean degree, 4.9; transitivity, 0.64) compared with the controls (mean degree, 3.6; transitivity, 0.58), suggesting that LC patients-enriched genera had a stronger correlation with each other than controls. The results indicated that patients-enriched species affected the host by interacting and exerting similar effects. Furthermore, degree centrality (DC), closeness centrality (CC) and betweenness centrality (BC) were used to screen the influential microbiota in each network (DC > 0.1, CC > 0.2, BC > 0.1). In LC patients, the roles of *Campylobacter, Atopobium, Haemophilus* and *Streptococcus* were several network-hubs and they were important to the lung microbial community alteration of the LC patients **(** **Figure 3A** **)**. Results in controls showed that *Bacillus, Fusobacterium, Alloprevotella, Klebsiella* and *Kroppenstedtia* contributed to more importance **(** **Figure 3B** **)**.

We constructed a correlation network combining the significantly different genera, which were obtained by 16S rRNA amplicon sequencing. *Lactobacillus, Brevundimonas, Massilia*, *Christensenellaceae R-7 group* were positively correlated with each other, which were enriched in controls, and they were negatively correlated with *Veillonella, Atopobium, Haemophilus*, *Fusobacterium*, which were enriched in LC patients **(** **Figure 3C** **)**. Based on the measurement indexes characterizing the properties of the networks (DC > 0.1, CC > 0.2, BC > 0.1), *Brevundimonas, Bacillus, Veillonella, Klebsiella* and *Pseudomonas* were identified.

### Identifying biomarkers in LC patients

**Figure 4:**
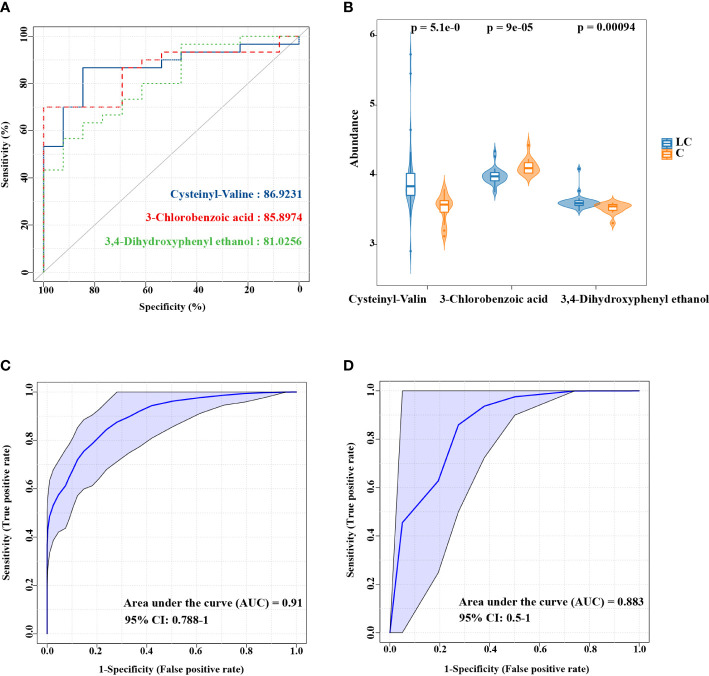
ROC curve analysis of the candidate biomarkers for LC. **(A)** Individual ROC curves and peak areas for Cysteinyl-Valine, 3-Chlorobenzoic acid and 3,4-Dihydroxyphenyl ethanol. **(B)** The differences in the abundance of three metabolic biomarkers between two groups. **(C)** ROC analysis based on the combination of three metabolites. **(D)** ROC analysis for the combination of 9 species screened by LASSO.

Due to the function of evaluating the predictive ability of models, ROC curve has been in widespread use. ROC curve was used to assess representative differential features for the diagnosis of LC in this study. As indicated by the results, the AUC of Cysteinyl-Valine, 3-Chlorobenzoic acid and 3,4-Dihydroxyphenyl ethanol were 0.8692, 0.859 and 0.8103 **(** **Supplementary Table S7** **)**, which might be useful in identifying patients with LC **(** **Figures 4A, B** **)**. In order to improve the accuracy of biomarkers, the three metabolites were combined for ROC analysis, which showed more strikingly capability of the diagnosis for LC (AUC:0.91, **Figure 4C**). LEfSe analysis based on 16S rRNA amplicon sequencing revealed 14 significantly different species (LDA > 2.0, *P* < 0.01), from which nine species were screened by LASSO **(** **Supplementary Figure S3**, **Supplementary Table S8** **)**. ROC analysis on the basis of the combination of the 9 species, demonstrating that LC could be assessed by representative differential lung microbiota **(** **Figure 4D** **)**.

## Discussion

LC tumor microenvironment is colonized by microbiota, which can interact with the host, and new studies have indicated that this might be a potential factor affecting LC. Generally speaking, the normal tissue microenvironment protects the lungs, while the tumor microenvironment promotes cancer progression. Therefore, we used the samples which could characterize the changes of lung microenvironment to explore the effects of the lung microbiota and metabolites on the progression of LC. This study suggested that the altered microbiota and metabolites between the patients with or without lung cancer might play pivotal roles in LC pathogenesis.

In the metabolomics analysis of flushing fluid samples, multiple fatty acyls were significantly upregulated in LC patients and glycerophospholipids accounted for the largest proportion in controls, which indicated that lipid metabolism changed in LC patients. Increasing evidences suggested that lipid metabolism could be assisted in determining tumor metastasis, improving therapeutic efficacy and developing new therapeutic targets (18). Lipids are components of cell membranes that are involved not only in energy storage but also as messengers in signaling. In addition, the disorder of lipid metabolism in cancer cells will affect cell proliferation and differentiation and other processes (19). As the main components of pulmonary surfactant, which is a complex of phospholipids (85% phosphatidylcholine) and surfactant proteins, lipids have been shown to play essential roles in the pathogenesis of LC (20, 21). Pulmonary surfactant was synthesized and secreted by alveolar type II cell, a type of lung stem cell and it could transform into monoclonal lung tumor during active KRAS mutation in previous studies (22). Various studies have shown that the destruction of pulmonary surfactant and the changes of alveolar type II cell homeostasis were connected with the pathogenesis of LC (23).

In particular, we found that metabolites of sphingosine enriched in sphingolipid signaling pathway, significantly decreased in LC patients. Sphingolipids are bioactive membrane lipids that act as first or second messengers (24, 25). In particular, the first sphingolipid detected was sphingosine, which could regulate various physiological processes such as cell cycle, apoptosis (26). Sphingosine, as a regulator that inhibits cell proliferation, can affect cell growth and apoptosis (27). Particularly, sphingosine is an important substance that helps protect the respiratory tract against bacterial pathogens (28). Sphingosine has been found to inhibit multiple pathogens, including *Staphylococcus aureus, Acinetobacter baumannii, Haemophilus influenzae, Escherichia coli, Fusobacterium nucleatum, Streptococcus sanguinis* (29). As the heat map showed, the bactericidal effect of sphingosine could have something to do with the downregulation of *Haemophilus* and *Streptococcus* in controls of our study.

Moreover, the pathway of ABC transporters, protein digestion and absorption and central carbon metabolism in patients were changed. Decreased level of ABC transporters was found in LC patients, containing betaine, L-Arginine and taurine. Betaine is widely regarded as an anti-oxidant and it has beneficial actions in several human diseases, such as obesity, diabetes and cancer (30). Tang et al. reported that choline-betaine pathway was conducive to hyperosmotic stress and lethal stress resistance in *Pseudomonas protegens SN15-2* (31), and this could have something to do with the enrichment of *Pseudomonas* in controls of our study. Arginine is present in the precursors of various organic compounds such as nitric oxide (NO), ornithine and myosine, which have huge impacts on immune cell biology, especially macrophage, dendritic cell and T cell immunobiology (32, 33). Kim et al. reported that arginine-induced changes in gut microbiota enhanced host lung immunity to nontuberculous mycobacterial infection, and that indicated that arginine might plays a protective role in lungs (34). Taurine, as conditionally essential amino acid of human, has multiple physiological functions, including the regulation of neural conduction, participating in endocrine activities, immunity enhancement, and strengthening the antioxidant capacity of cytomembrane (35). Taurine was found to inhibit the proliferation of lung cancer cells, significantly boosted the apoptosis rate, and reduced the expression of migration factors matrix metallopeptidase 9 (MMP-9) and vascular endothelial growth factor (VEGF) (36, 37). Previous studies have shown that taurine ABC transporter protein has been identified in *Lactobacillus*, and this could have something to do with the enrichment of *Lactobacillus* in controls of our study (38). The upregulation of betaine, arginine and taurine in controls might contribute to the immunity enhancement and the boost of the antioxidant capacity of cells. The pathway of protein digestion and absorption and central carbon metabolism in cancer contained a variety of amino acids such as L-Tryptophan with decreased relative abundance in LC patients. Tryptophan is an essential amino acid and plays essential roles in various physiological processes. Down-regulated tryptophan concentration have been detected in patients with colorectal cancer, malignant melanoma and LC, and studies showed that tryptophan metabolites could drive the motility and migration of cancer cells (39). In addition, the pathway of Linoleic acid metabolism involved in metabolites of 9, 10-DHOME was up-regulated in LC. The level of 9, 10-DHOME, which was the epoxide hydrolase metabolite of the leukotoxin 9,10-EpOME, was found to be increased in disease. 9, 10-DHOME activates the NF-κB and AP-1 transcription factors of endotheliocyte to mediate inflammatory responses (40). Moreover, many studies showed that DiHOMEs might be part of the inflammatory response to environmental insults in lungs (41).

In this research, we explored the microbial changes in BALF samples using 16S rRNA amplicon sequencing. Results showed that the microbiota constitution in LC patients was different from that of controls and the microbiota differed in terms of beta-diversity. The microbial dysbiosis of LC patients was represented by decreasing microbial diversity, and increasing *Streptococcus*, *Prevotella*, *Veillonella* and *Haemophilus*, which were in accordance with the existing results (9, 42). Elevated abundance of *Streptococcus*, *Prevotella* and *Veillonella* were found in tumor tissues from LC patients previously, and the changes of these genera were related to the up-regulation of ERK and PI3K signaling pathways in LC patients (9). We also found that *Fusobacterium* was up-regulated in LC patients. The promotion effect of *Fusobacterium* on tumor cells is mainly achieved by inhibiting host immunity and inducing proinflammatory microenvironment (43). The available studies demonstrated that *Fusobacterium* acts as an inducer in various cancers, such as breast, colon and oral cancer (44–46). Several studies on the mechanism of *Fusobacterium* in promoting tumor development had provided different results. High levels of *Fusobacterium* promoted the activity of NF-κB and various pro-inflammatory factors, and the FadA virulence factor in *Fusobacterium* affected cell growth by regulating the β-catenin signaling pathway (47, 48).

LEfSe analysis showed that potential probiotics, including *Lactobacillus*, *Lactococcus*, Oscillospirales and Christensenellaceae, were down-regulated in LC patients. Probiotics were found to have the ability to achieve anticancer effects by promoting apoptosis of cancer cells and improving resistance to oxidative stress (49, 50). Multiple common microorganisms in the human gut have probiotic effects such as *Bifidobacterium*, *Lactobacillus*, *Lactococcus*. In particular, many lactic acid bacteria (LAB) have essential conducive impacts on the host such as anti-oxidation and anti-inflammation (51). The antioxidant capacity of LAB is based on the high catalase and α, α-diphenyl-β-picrylhydrazyl (DPPH) free radical scavenging activity, the anti-inflammatory property is achieved by the promotion of anti-inflammatory cytokines (IL-10) as well as the decrease of proinflammatory cytokines (IL-6) (43). Oscillospirales was believed to produce short-chain fatty acids, and the level of it was also found to be decreased in disease (52). The Christensenellaceae has been found in human bodies, which plays an important role in human health (53).

The correlation networks showed that multiple oral bacteria were enriched in the lungs, and there was a strong correlation between them such as *Veillonella, TM7x, Capnocytophaga, Parvimonas, Granulicatella*. There has been an increasing interest in detecting the connection between oral microbiota and the occurrence of respiratory tract infections. Associations between oral microbiota and several respiratory infections have been reported previously (54). A previous study found that oral commensal microbiota was enriched in the lower airway of LC patients, and the connections between the lower airway microbiota and host immunity in healthy subjects have also been explored (9). Previous studies confirmed that distinguishing oral commensal microbiotas were detected to have changes during the development of cancers such as pancreatic cancer, breast cancer or LC (55–57). However, none of them clearly elucidated the relationships between oral commensal microbiota and the pathogenesis of multiple cancers.

However, our results still have some shortcomings, and did not consider the tumor stage, the histological subtype and clinical validation. Many studies have found differences in the characteristics of microbiota between different tumor stages and histological subtypes in other cancers (58, 59). In the subsequent studies, we will expand sample size, and evaluate the potential marker in a larger cohort. We hope to verify the diagnostic value of the biomarkers and explore the molecular mechanisms by which lung microbiota and metabolites affect LC. Moreover, the relationship between lung microbiota and metabolites in different tumor stages and histological subtypes will be considered.

## Conclusions

In this study, the differences in lung microbiota and metabolites between LC patients and patients with non-lung cancer were explored by 16S rRNA amplicon sequencing, metagenomics and metabolomics. The results suggested that lung microbiota and metabolites might play critical roles in the progression of LC. The composition of the lung metabolites was significantly different between the LC patients and controls, which indicated that lipid metabolism, especially sphingolipid signaling pathway, changed in LC patients. The microbiota in LC patients were different from those in controls, with multiple probiotics were down-regulated in LC patients. Moreover, we found that oral commensal microbiota might be related to the development and progression of LC. Finally, we found 3 metabolites and 9 species, which have significantly differences, and they might have the potential to be clinical diagnostic markers and therapeutic targets associated with LC.

## Data availability statement

The datasets presented in this study can be found in online repositories. The names of the repository/repositories and accession number(s) can be found below: https://www.ncbi.nlm.nih.gov/, PRJNA858501, https://www.ncbi.nlm.nih.gov/, PRJNA858534.

## Ethics statement

The studies involving human participants were reviewed and approved by Ethics Committee of Zibo Municipal Hospital (20201102). The patients/participants provided their written informed consent to participate in this study. Written informed consent was obtained from the individual(s) for the publication of any potentially identifiable images or data included in this article.

## Author contributions

MS received the research grant, conceived and designed this study and revised the manuscript. BL and YL analyzed clinical and microbiome data and wrote the manuscript. LS enrolled the study participants, and revised the manuscript. WZ, RW, HC, JL, XY, SX and WW performed the experiments. LD and SL supervised and provided continuous guidance for the experiments. All authors approved the final version of the manuscript before submission.

## Funding

This work was supported by the National Key Research and Development Program of China (grant no. 2021YFA0717002), Taishan Scholars Program of Shandong Province (grant no. tsqn202103196), and the Fundamental Research Funds for the Central Universities (grant no. 202172002).

## Conflict of interest

The authors declare that the research was conducted in the absence of any commercial or financial relationships that could be construed as a potential conflict of interest.

## Publisher’s note

All claims expressed in this article are solely those of the authors and do not necessarily represent those of their affiliated organizations, or those of the publisher, the editors and the reviewers. Any product that may be evaluated in this article, or claim that may be made by its manufacturer, is not guaranteed or endorsed by the publisher.
