# Supplementary material for: Characterizing microbiota and metabolomics analysis to identify candidate biomarkers in lung cancer
Source: Front Oncol. 2022 Nov 15;12:1058436. doi: 10.3389/fonc.2022.1058436 (PMC9705781; doi:10.3389/fonc.2022.1058436)
Supplement: Supplementary file 1 [file DataSheet_1.pdf]

Supplementary Material

1 Supplementary Tables

Supplementary Table 1. Demographic and clinical characteristics of the subjects participating in 16S rRNA amplicon sequencing.

Patient characteristics	All Subjects (n= 16)	Cancer group (n =7)	Control group (n = 9)
Age (mean ± S.D.)	64.8 ± 4.0	64.0 ± 3.3	65.3 ± 4.6
Gender (n, %)			
Male	11 (68.7)	5 (71.4)	6 (66.7)
Female	5 (31.3)	2 (28.6)	3 (33.3)
Diagnosis (n, %)			
Lung cancer			
Adeno	3 (18.8)	1 (14.3)	2 (22.3)
Squamous	3 (18.8)	1 (14.3)	2 (22.3)
Small cell	4 (25.0)	2 (28.6)	2 (22.3)
Carcinoma NOS‡	6 (37.5)	3 (42.8)	3 (33.1)
Tumor stage (n, %)			
I	3 (18.8)	1 (14.3)	2 (22.2)
II	0	0	0
III	4 (25.0)	2 (28.6)	2 (22.2)
IV	2 (12.5)	1 (14.3)	1 (11.1)
Unknown	7 (43.7)	3 (42.8)	4 (44.5)
History of diseases			

Hypertension			
Yes	1 (6.3)	1 (14.3)	0
NO	15 (93.7)	6 (85.7)	9 (100.0)
Diabetes			
Yes	1 (6.3)	0	1 (11.1)
NO	15 (93.7)	7 (100.0)	8 (88.9)

Cancer group: samples of the bronchoalveolar lavage fluid from the cancer lobes.

Control group: samples of the bronchoalveolar lavage fluid from the contralateral non-lung cancer lobes in the same patients.

Definition of abbreviations: NOS = not otherwise specified

Data are presented as % (n) or mean ± S.D.

‡ NOS indicates clinically diagnosed as non-lung cancer; however, no specific diagnosis made.

Supplementary Table 2. Demographic and clinical characteristics of the subjects participating in metagenomics sequencing.

Patient characteristics	All Subjects (n= 12)	Cancer group (n =7)	Control group (n = 5)
Age (mean ± S.D.)	63.9 ± 2.7	64.0 ± 3.3	63.8 ± 1.9
Gender (n, %)			
Male	8 (66.7)	5 (71.4)	3 (60.0)
Female	4 (33.3)	2 (28.6)	2 (40.0)
Diagnosis (n, %)			
Lung cancer			
Adeno	3 (25.0)	1 (14.3)	2 (40.0)

Squamous	2 (16.7)	1 (14.3)	1 (20.0)
Small cell	2 (16.7)	2 (28.6)	0
Carcinoma NOS‡	5 (41.6)	3 (42.8)	2 (40.0)
Tumor stage (n, %)			
I	2 (16.7)	1 (14.3)	1 (20.0)
II	0	0	0
III	3 (25.0)	2 (28.6)	1 (20.0)
IV	1 (8.3)	1 (14.3)	0
Unknown	6 (50.0)	3 (42.8)	3 (60.0)
History of diseases			
Hypertension			
Yes	1 (8.3)	1 (14.3)	0
NO	11 (91.7)	6 (85.7)	5 (100.0)
Diabetes			
Yes	1 (8.3)	0	1 (20.0)
NO	11 (91.7)	7 (100.0)	4 (80.0)

Cancer group: samples of the bronchoalveolar lavage fluid from the cancer lobes.

Control group: samples of the bronchoalveolar lavage fluid from the contralateral non-lung cancer lobes in the same patients.

Definition of abbreviations: NOS = not otherwise specified

Data are presented as % (n) or mean ± S.D.

‡ NOS indicates clinically diagnosed as non-lung cancer; however, no specific diagnosis made.

Supplementary Table 3. Demographic and clinical characteristics of the subjects participating in metabolomics analysis.

Patient characteristics	All Subjects (n= 43)	Cancer group (n =30)	Control group (n = 13)
Age (mean ± S.D.)	59.1 ± 11.9	59.0 ± 12.9	59.5 ± 9.5
Gender (n, %)			
Male	18 (41.9)	12 (40.0)	6 (46.2)
Female	25 (58.1)	18 (60.0)	7 (53.8)
Diagnosis (n, %)			
Lung cancer			
Adeno	30 (69.8)	30 (100.0)	
Non–lung cancer			
Sarcoidosis	1 (2.3)		1 (2.3)
Carcinoid	11 (25.6)		11 (25.6)
Benign NOS	1 (2.3)		1 (2.3)
Tumor stage (n, %)			
I	28 (93.3)	28 (93.3)	
Unknown	2 (6.7)	2 (6.7)	
History of diseases			
Hypertension			
Yes	7 (16.3)	4 (13.3)	3 (23.1)
NO	36 (83.7)	26 (86.7)	10 (76.9)
Diabetes			
Yes	0	0	0
NO	43 (100.0)	30 (100.0)	13 (100.0)

Cancer group: samples of the lung tissue flushing solutions from the tumors of lung

cancer patients.

Control group: samples of the lung tissue flushing solutions from the tissues of non-lung cancer patients.

Definition of abbreviations: NOS = not otherwise specified

Data are presented as % (n) or mean ± S.

Supplementary Table 4. Metabolites with differential relative abundance between the lung cancer patients and controls.

Metabolite	FC (LC/C)	VIP	P_value
Cysteinyl-Valine	1.122263293	1.059156238	0.0001478
3-Chlorobenzoic acid	0.966504854	2.039767300	0.0002252
3,4-Dihydroxyphenyl ethanol	1.025284091	1.213566060	0.0014400
Melilotoside C	0.816638370	2.016594913	0.0026870
PC (18:0/20:4(5Z,8Z,11Z,14Z))	0.866699826	3.064063186	0.0034770
9,10-DHOME	1.279329609	2.155802529	0.0034770
PE(O-18:1(1Z)/20:4(5Z,8Z,11Z,14Z))	0.892729440	2.667764217	0.0041150
PE (18:0/20:4(5Z,8Z,11Z,14Z))	0.967664409	1.577385251	0.0062010
Goyaglycoside g	0.860914482	1.985512885	0.0078690
Pseudoephedrine	1.164635603	1.513250050	0.0085080
4-hydroxy-3,5,6-trimethylpyran-2-one	1.126047359	1.179712048	0.0091940
PE-NMe (15:0/20:4(5Z,8Z,11Z,14Z))	0.904171934	2.470642070	0.0115600
PE (18:4(6Z,9Z,12Z,15Z)/20:5(5Z,8Z,11Z,14Z,17Z))	0.863552777	2.081250608	0.0144400
N-Isobutyl-2,4,8,10,12-tetradecapentaenamide	1.055777731	1.179832163	0.0167000

2-Pentadecylfuran	0.932607990	2.229493409	0.0167000
4-Hydroxy-3-methoxy-2,10-bisaboladien-9-one	1.041501191	1.062353118	0.0167000
4-Hydroxyhippuric acid	1.057362507	1.653848014	0.0192700
Hexaethylene glycol	0.786442285	1.929503894	0.0192700
Avocadene	1.029426031	1.040024499	0.0192700
PC (18:0/18:2(9Z,12Z))	0.865465606	2.975482851	0.0221700
P-Acetaminobenzoic acid	0.964364709	1.279251775	0.0237600
PE (18:4(6Z,9Z,12Z,15Z)/18:4(6Z,9Z,12Z,15Z))	0.826689446	2.155798732	0.0237600
Sphingosine	0.961416930	1.316803329	0.0254500
PC (14:0/16:0)	0.821274298	3.328173874	0.0254500
Rotundine A	1.040701451	1.109220265	0.0254500
2-Propenyl phenylacetate	1.058975685	1.000933309	0.0272400
PE (15:0/22:1(13Z))	0.913887072	2.439433411	0.0291400
Erucic acid	1.041034971	1.478232508	0.0311500
Serratol	0.928148441	2.204956393	0.0311500
3beta-7-Drimene-3,11-diol	1.060992908	1.123088218	0.0355300
PE (14:1(9Z)/14:1(9Z))	0.803113553	2.114971067	0.0355300
Polyethylene, oxidized	1.101019758	1.096910092	0.0355300
3,4-Dihydro-2,2,5,7,8-pentamethyl-2H-1-benzopyran-6-ol	0.846938776	2.968294944	0.0379100
Heptaethylene glycol	0.905970149	1.945050522	0.0379100

Glabric acid	0.628488372	1.252873099	0.0404200
4,5-Dihydroorotic acid	0.631827112	3.096603668	0.0404200
Lysinoalanine	0.585004359	3.205209055	0.0404200
4-Hydroxy-alprenolol	0.888381086	1.756537792	0.0404200
7-Methylguanosine	0.874451273	1.103279021	0.0488300
PE (18:0/0:0)	0.859941804	3.065421059	0.0488300

LC, lung cancer; C, control; VIP, variable importance in projection.

Supplementary Table 5. Microbial composition at phylum and genus levels of lung cancer patients and controls based on 16S rRNA amplicon sequencing.

Phylum level			Genus level		
TAXA	Percentage		TAXA	Percentage	
	LC (%)	C (%)		LC (%)	C (%)
Proteobacteria	45.05	45.26	*Pseudomonas*	35.14	36.25
Firmicutes	28.31	28.82	*Streptococcus*	14.34	12.52
Bacteroidota	14.89	10.63	*Prevotella*	9.550	6.400
Actinobacteriota	7.15	9.28	*Neisseria*	6.810	3.990
Fusobacteriota	2.41	3.74	*Actinomyces*	4.600	4.610
Patescibacteria	1.25	0.93	*Veillonella*	4.850	3.160
others	0.94	1.34	*Granulicatella*	3.530	2.920
			Alloprevotella	3.250	2.100

Leptotrichia	1.270	2.780
Rhodococcus	0.910	2.390
Fusobacterium	1.130	0.900
Klebsiella	0.050	1.910
Haemophilus	1.070	0.660
Porphyromonas	1.120	0.570
Lactobacillus	0.120	1.320
Bacillus	0.110	1.160
others	12.15	16.36

LC, lung cancer; C, control.

Supplementary Table 6. COG functional categories with differences between the lung cancer patients and controls.

Name	C-Mean (%)	C-Sd (%)	LC-Mean (%)	LC-Sd (%)	P_value
COG2202	0.05486	0.01861	0.004417	0.008129	0.004200
COG2081	0.05420	0.06999	0.002225	0.003930	0.004200
ENOG410XNWU	0.04587	0.01407	0.007747	0.015300	0.007054
ENOG4110ZRH	0.03771	0.02436	0.002442	0.004212	0.007054
COG2060	0.08916	0.03602	0.019700	0.024960	0.008168
COG2812	0.07020	0.04031	0.012490	0.017890	0.008168
ENOG410XP3U	0.04350	0.01955	0.008727	0.012140	0.008168

COG1937	0.03005	0.02695	0	0	0.009088
ENOG4111STD	0.02677	0.02936	0	0	0.009088
COG0229	0.02200	0.01391	0	0	0.009088
COG3105	0.02141	0.02018	0	0	0.009088
ENOG410XR7F	0.02016	0.02668	0	0	0.009088
ENOG410XPWE	0.01540	0.01199	0	0	0.009088
ENOG410YW9K	0.01487	0.01107	0	0	0.009088
COG0750	0.05416	0.05731	0.003116	0.008243	0.009314
COG0006	0.04656	0.02334	0.008477	0.016010	0.01154
ENOG410XZDR	0.04010	0.01996	0.005715	0.011970	0.01154
ENOG4111NYG	0.02261	0.007218	0.003669	0.007068	0.01154
COG0169	0.07262	0.037530	0.009419	0.013010	0.01315
COG0063	0.02189	0.005573	0.006321	0.008166	0.01315
COG0087	0.009688	0.01761	0.162000	0.152200	0.01468
ENOG410Y32P	0.07082	0.09375	0.001450	0.003837	0.01856
COG0176	0.06351	0.07648	0.001104	0.002922	0.01856
COG2193	0.04590	0.04193	0.003256	0.008616	0.01856
COG1270	0.04312	0.03023	0.002134	0.005647	0.01856
COG2423	0.04102	0.03019	0.001357	0.003591	0.01856
COG2085	0.03962	0.03490	0.001127	0.002982	0.01856
COG5478	0.03495	0.03299	0.001535	0.004062	0.01856

COG0216	0.03160	0.02010	0.003289	0.008702	0.01856
COG1154	0.03298	0.02173	0.001126	0.002978	0.01856
COG2832	0.03044	0.02319	0.001904	0.005038	0.01856
ENOG410YTAS	0.03047	0.01855	0.001551	0.004104	0.01856
COG2334	0.02638	0.01818	0.001235	0.003267	0.01856
COG1942	0.02503	0.02128	0.001618	0.004281	0.01856
COG3745	0.02060	0.01553	0.001546	0.004092	0.01856
COG0476	0.02048	0.01391	0.001231	0.003256	0.01856
COG5042	0.01634	0.009544	0.0009702	0.002567	0.01856
ENOG410ZRA3	0.01425	0.008449	0.0008739	0.002312	0.01856
COG3508	0.009975	0.006181	0.0009375	0.002480	0.01856
ENOG4111IA5	0.009732	0.006518	0.0006543	0.001731	0.01856
ENOG410Y4E6	0.001958	0.002687	0.1119000	0.124900	0.02563
ENOG4111QFM	0.036300	0.03479	0.0025350	0.006706	0.02977
COG0325	0.03092	0.03391	0.0014750	0.003902	0.02977
COG0128	0.02653	0.03720	0.0016730	0.004427	0.02977
COG0594	0.02548	0.02306	0.0015800	0.004180	0.02977
COG5345	0.01908	0.01878	0.0017010	0.004501	0.02977
ENOG410ZHXN	0.01316	0.01179	0.0013320	0.003525	0.02977
ENOG410XQTE	0.01289	0.01141	0.0007837	0.002073	0.02977

COG1541	0.009614	0.006972	0.0012990	0.003436	0.02977
COG0246	0.05779	0.05573	0.0056750	0.010920	0.03024
ENOG410YC0E	0.05703	0.05795	0.0027790	0.004794	0.03024
COG0016	0.04809	0.05184	0.0052770	0.009494	0.03024
COG0684	0.04694	0.03732	0.0055610	0.012140	0.03024
COG0804	0.04938	0.04239	0.0013140	0.002266	0.03024
COG0392	0.03866	0.02427	0.0081010	0.013860	0.03024
COG1366	0.03832	0.02489	0.0055580	0.009623	0.03024
COG1381	0.03926	0.03145	0.0041430	0.007740	0.03024
COG3652	0.03551	0.02541	0.0027130	0.004680	0.03024
COG3138	0.03567	0.03480	0.0019310	0.003410	0.03024
COG0302	0.02994	0.02413	0.0039060	0.007505	0.03024
ENOG4111Y3P	0.02302	0.02071	0.002518	0.004447	0.03024
ENOG4111WIR	0.02173	0.01916	0.002062	0.003557	0.03024
COG4757	0.01770	0.01309	0.003540	0.006061	0.03024
COG0649	0.01651	0.01050	0.002168	0.003720	0.03024
ENOG410XNPX	0.01533	0.01027	0.002557	0.004387	0.03024
COG0845	0.18100	0.13370	0.035490	0.048080	0.03163
COG3842	0.15080	0.08127	0.051490	0.067940	0.03163
COG0085	0.06907	0.03830	0.018260	0.025140	0.03163
ENOG410Y01S	0.04872	0.01536	0.015190	0.019570	0.03163

COG3299	0.02819	0.01965	0.007617	0.011740	0.03163
COG0099	0.09077	0.18530	0	0	0.03304
ENOG410ZU0E	0.04208	0.07423	0	0	0.03304
ENOG410XR6Z	0.04011	0.08480	0	0	0.03304
ENOG410XWE8	0.03675	0.06008	0	0	0.03304
ENOG4111QUR	0.03061	0.05264	0	0	0.03304
COG3826	0.02985	0.04943	0	0	0.03304
ENOG4111MUY	0.02730	0.05085	0	0	0.03304
ENOG410Y3ZC	0.02719	0.05212	0	0	0.03304
ENOG410ZXVQ	0.02522	0.04587	0	0	0.03304
COG1551	0.02323	0.03400	0	0	0.03304
ENOG4111KE4	0.02313	0.02999	0	0	0.03304
COG3063	0.02311	0.03846	0	0	0.03304
ENOG411227T	0.02285	0.03598	0	0	0.03304
COG0284	0.02232	0.02759	0	0	0.03304
COG2198	0.02160	0.04186	0	0	0.03304
COG1261	0.02079	0.02980	0	0	0.03304
ENOG4111HFM	0.02039	0.03477	0	0	0.03304
COG3190	0.02020	0.03011	0	0	0.03304
COG0071	0.02010	0.03362	0	0	0.03304

COG0316	0.01860	0.01980	0	0	0.03304
COG3816	0.01843	0.02054	0	0	0.03304
ENOG410XVCJ	0.01838	0.02986	0	0	0.03304
ENOG410XUF0	0.01790	0.02992	0	0	0.03304
COG2860	0.01781	0.02655	0	0	0.03304
COG0263	0.01770	0.02733	0	0	0.03304
COG0720	0.01731	0.02701	0	0	0.03304
COG3549	0.01721	0.02780	0	0	0.03304
ENOG410ZVCU	0.01719	0.01667	0	0	0.03304
COG1559	0.01717	0.01777	0	0	0.03304
COG3636	0.01705	0.02511	0	0	0.03304
ENOG41121V4	0.01694	0.02418	0	0	0.03304
ENOG410ZCZ7	0.01653	0.01741	0	0	0.03304
ENOG410XT0W	0.01649	0.01809	0	0	0.03304
ENOG410ZAGI	0.01634	0.02420	0	0	0.03304
ENOG410ZZE3	0.01614	0.02758	0	0	0.03304
COG3817	0.01598	0.01555	0	0	0.03304
COG3098	0.01593	0.02897	0	0	0.03304
COG3126	0.01573	0.01758	0	0	0.03304
ENOG410Y3MJ	0.01571	0.02473	0	0	0.03304
ENOG4112DFI	0.01553	0.02354	0	0	0.03304

ENOG410Y8BR	0.01526	0.02343	0	0	0.03304
ENOG4111UJ7	0.01519	0.02360	0	0	0.03304
COG1943	0.01513	0.01624	0	0	0.03304
ENOG4111JN5	0.01479	0.02360	0	0	0.03304
ENOG41125Z4	0.01475	0.02173	0	0	0.03304
ENOG4111VYG	0.01458	0.02487	0	0	0.03304
COG0690	0.01385	0.02098	0	0	0.03304
COG3558	0.01327	0.01711	0	0	0.03304
ENOG410XQX0	0.01261	0.01487	0	0	0.03304
ENOG4111QYX	0.01249	0.01353	0	0	0.03304
ENOG41129S9	0.01226	0.01531	0	0	0.03304
COG3147	0.01197	0.01241	0	0	0.03304
COG0565	0.01185	0.01193	0	0	0.03304
ENOG410Y91C	0.011800	0.01229	0	0	0.03304
ENOG4111YWN	0.011200	0.01379	0	0	0.03304
COG0830	0.011130	0.01594	0	0	0.03304
ENOG4112C2F	0.010880	0.01226	0	0	0.03304
ENOG410ZVYD	0.010810	0.01219	0	0	0.03304
ENOG4111IMG	0.010240	0.01430	0	0	0.03304
COG0483	0.010150	0.01312	0	0	0.03304

COG4445	0.009488	0.01237	0	0	0.03304
COG4525	0.008435	0.01114	0	0	0.03304
ENOG410ZM68	0.007929	0.009749	0	0	0.03304
COG2957	0.007870	0.007625	0	0	0.03304
COG0753	0.109500	0.048350	0.04064	0.04054	0.03444
COG1629	0.531100	0.172400	0.28380	0.21950	0.03476
COG2032	0.156300	0.253100	0.03173	0.07637	0.04331
COG2909	0.095970	0.076670	0.02280	0.03382	0.04331
COG2007	0.009723	0.021740	0.10120	0.10780	0.04331
COG4174	0.059300	0.041640	0.01031	0.01448	0.04331
COG2025	0.042700	0.023960	0.01435	0.01936	0.04331
COG3023	0.043270	0.026100	0.01212	0.01823	0.04331
ENOG410XS4R	0.030750	0.013000	0.009405	0.01610	0.04331
COG1951	0.025010	0.018580	0.005273	0.007387	0.04331
COG3568	0.02411	0.016630	0.004970	0.006945	0.04331
COG1866	0.02009	0.012640	0.004092	0.005962	0.04331
ENOG410Y742	0.01635	0.009642	0.004080	0.006034	0.04331
COG1940	0.08383	0.123200	0.005142	0.009595	0.0462
COG0771	0.06684	0.070290	0.008849	0.019500	0.0462
ENOG4111R1A	0.05416	0.044160	0.006561	0.013490	0.0462
COG0349	0.04677	0.036470	0.006846	0.012760	0.0462

COG0407	0.04685	0.064520	0.002407	0.004558	0.0462
COG0066	0.03963	0.034770	0.007166	0.012290	0.0462
ENOG4111JPP	0.03391	0.032640	0.005009	0.009856	0.0462
COG3520	0.02791	0.020700	0.005420	0.009341	0.0462
COG3495	0.02274	0.014580	0.004229	0.008084	0.0462
COG0367	0.02277	0.017260	0.004164	0.008339	0.0462
COG2064	0.02189	0.017720	0.003090	0.005330	0.0462
ENOG4111HFG	0.01454	0.008983	0.002378	0.004569	0.0462
COG4590	0.01243	0.01459	0.001957	0.003562	0.0462
COG1959	0.02971	0.02708	0.004477	0.011840	0.04637
COG2026	0.02791	0.02218	0.004834	0.012790	0.04637
ENOG4112B17	0.02703	0.03506	0.004177	0.011050	0.04637
COG2022	0.02562	0.02784	0.003332	0.008815	0.04637
ENOG410Z4H2	0.02257	0.01965	0.004207	0.011130	0.04637
ENOG410Y20W	0.01859	0.01662	0.003002	0.007942	0.04637
ENOG410XRKR	0.01835	0.01722	0.001537	0.004067	0.04637
COG0455	0.01498	0.01012	0.002402	0.006354	0.04637
COG0765	0.11010	0.05556	0.038800	0.05094	0.04728
COG1253	0.09244	0.06417	0.027080	0.04485	0.04728
COG0778	0.08615	0.05134	0.028870	0.04951	0.04728

COG0637	0.08356	0.06360	0.029200	0.04822	0.04728
COG2391	0.05558	0.02154	0.022440	0.03946	0.04728
COG4799	0.05477	0.06137	0.010760	0.01758	0.04728
COG0280	0.02849	0.003827	0.014140	0.02495	0.04728
COG0697	0.13570	0.061460	0.051690	0.06005	0.04970
COG0404	0.08549	0.037000	0.038870	0.03759	0.04970
COG3659	0.05567	0.030260	0.020850	0.02881	0.04970
COG1593	0.03345	0.013710	0.013590	0.01416	0.04970

COG, clusters of orthologous groups; LC, lung cancer; C, control; SD, standard deviation.

Supplementary Table 7. AUC values of differential metabolites evaluated by ROC curves.

Metabolite	Metab ID	AUC	CI
Cysteinyl-Valine	metab_02687	0.8692	[0.7590, 0.9795]
3-Chlorobenzoic acid	metab_15183	0.8590	[0.7476, 0.9704]
3,4-Dihydroxyphenyl ethanol	metab_16109	0.8103	[0.6769, 0.9436]
Melilotoside C	metab_08729	0.7923	[0.6573, 0.9273]
PC (18:0/20:4 (5Z,8Z,11Z,14Z))	metab_14495	0.7846	[0.6428, 0.9265]
9,10-DHOME	metab_04107	0.7846	[0.6085, 0.9607]
PE (O-18:1 (1Z)/20:4 (5Z,8Z,11Z,14Z))	metab_14448	0.7795	[0.6369, 0.9221]
PE (18:0/20:4 (5Z,8Z,11Z,14Z))	metab_13497	0.7667	[0.6220, 0.9113]
Goyaglycoside g	metab_08480	0.7590	[0.6127, 0.9053]

Pseudoephedrine	metab_00675	0.7564	[0.5965, 0.9163]
4-hydroxy-3,5,6-trimethylpyran-2-one	metab_04045	0.7538	[0.6019, 0.9058]
PE-NMe (15:0/20:4 (5Z,8Z,11Z,14Z))	metab_14527	0.7462	[0.5920, 0.9003]
PE (18:4 (6Z,9Z,12Z,15Z)/20:5 (5Z,8Z,11Z,14Z,17Z))	metab_03607	0.7385	[0.5827, 0.8943]
2-Pentadecylfuran	metab_04829	0.7333	[0.5760, 0.8906]
N-Isobutyl-2,4,8,10,12-tetradecapentaenamide	metab_03668	0.7333	[0.5675, 0.8991]
4-Hydroxy-3-methoxy-2,10-bisaboladien-9-one	metab_07366	0.7333	[0.5741, 0.8926]
Hexaethylene glycol	metab_02954	0.7282	[0.5750, 0.8815]
Avocadene	metab_04214	0.7282	[0.5613, 0.8951]
4-Hydroxyhippuric acid	metab_16097	0.7282	[0.5164, 0.9400]
PC (18:0/18:2 (9Z,12Z))	metab_14474	0.7231	[0.5609, 0.8852]
P-Acetaminobenzoic acid	metab_15385	0.7205	[0.5393, 0.9017]
PE (18:4(6Z,9Z,12Z,15Z)/18:4 (6Z,9Z,12Z,15Z))	metab_08510	0.7205	[0.5440, 0.8971]
Rotundine A	metab_09253	0.7179	[0.5474, 0.8885]
Sphingosine	metab_00454	0.7179	[0.5229, 0.9130]
PC (14:0/16:0)	metab_01543	0.7179	[0.5534, 0.8825]
2-Propenyl phenylacetate	metab_07903	0.7154	[0.5473, 0.8835]
PE (15:0/22:1 (13Z))	metab_14484	0.7128	[0.5518, 0.8738]
Serratol	metab_06520	0.7103	[0.5346, 0.8859]
Erucic acid	metab_04733	0.7103	[0.5449, 0.8757]

PE (14:1(9Z)/14:1 (9Z))	metab_03420	0.7051	[0.5365, 0.8738]
3beta-7-Drimene-3,11-diol	metab_13157	0.7051	[0.5250, 0.8853]
Polyethylene, oxidized	metab_07802	0.7051	[0.5297, 0.8805]
Heptaethylene glycol	metab_00015	0.7026	[0.5391, 0.8660]
3,4-Dihydro-2,2,5,7,8-pentamethyl-2H-1-benzopyran-6-ol	metab_12920	0.7026	[0.5227, 0.8824]
4,5-Dihydroorotic acid	metab_12169	0.7000	[0.5255, 0.8745]
Glabric acid	metab_11393	0.7000	[0.5406, 0.8594]
Lysinoalanine	metab_02468	0.7000	[0.5364, 0.8636]
4-Hydroxy-alprenolol	metab_09242	0.7000	[0.5329, 0.8671]
7-Methylguanosine	metab_16205	0.6923	[0.5278, 0.8568]

ROC, receiver operating characteristic;AUC, area under curve; CI, concordance index.

Supplementary Table 8. Nine species with significant differences were screened by LASSO.

Species name	group	Mean	LDA_value	P_value
s__Lactobacillus_murinus	C	2.583469085	2.234715054	0.011572728
s__uncultured_bacterium_g__norank_f__Muribaculaceae	C	3.270990796	2.937331316	0.011572728
s__Pelomonas_saccharophila	C	3.953107231	3.595062977	0.011572728
s__uncultured_Olsenella_sp__g__Atopobium	C	2.736602022	2.328692263	0.017514422
s__Corynebacterium_glutamicum	C	2.174805211	2.341196336	0.022934368
s__uncultured_rumen_bacterium_g__Prevotella	C	2.669655232	2.328451829	0.024308810

s__Massilia_timonae	C	2.815783268	2.512879658	0.024308810
s__Lactobacillus_delbrueckii_subsp__bulgaricus	C	2.137016650	2.253206474	0.027470761
s__Lactobacillus_reuteri	C	2.779121282	2.352214830	0.029727343

LASSO, least absolute shrinkage and selection operator; C, control; LDA, linear discriminant analysis.

2 Supplementary Figure legends

Supplementary Figure 1. Evaluation for the data quality of metabolome. **(A)** Relative standard deviation (RSD) curve was used to evaluate the stability of samples in the ESI+. **(B)** Relative standard deviation (RSD) curve was used to evaluate the stability of samples in the ESI-. **(C)** Principal component analysis (PCA) evaluated the data quality of QC samples in ESI+. **(D)** Principal component analysis (PCA) evaluated the data quality of QC samples in ESI-. **(E)** Compounds were classified by comparison with the KEGG Compound database. **(F)** The functions of metabolic pathways were annotated by the KEGG database, and the significantly different pathways ($P < 0.01$) were displayed by a bubble plot.

Supplementary Figure 2. Bacteria characteristics of BALF samples. **(A)** The species cumulative curve at the OTU classification level. **(B)** The rarefaction curve at the OTU classification level. **(C)** Venn diagram showed the differences of OTUs between the data sets of LC patients (blue circle) and controls (yellow circle). **(D)** Species alpha-diversity was measured using the Shannon index, Chao index and Ace index at the genus classification level. **(E)** A heat map to show the association between 20 differential genera and 40 differential metabolites closely related with progression of lung cancer. Each row represents a differential microorganism at the genus level, and each column represents a differential metabolite. Yellow means enriched in LC group, blue means enriched in control group, and gray means the genus and the metabolite had different trends in LC group. **(F)** Linear regression analysis of the consistency between species and functional diversity ($R^2 > 0.8$, $P < 0.01$).

Supplementary Figure 3. The box plots showed the differences in the abundance of 9 species between LC patients (red) and controls (blue), which were revealed by the LEfSe analysis of 16S rRNA amplicon sequencing and screened by LASSO.
