# Supplementary figures and images for: Characterizing microbiota and metabolomics analysis to identify candidate biomarkers in lung cancer
Source: Front Oncol. 2022 Nov 15;12:1058436. doi: 10.3389/fonc.2022.1058436 (PMC9705781; doi:10.3389/fonc.2022.1058436)

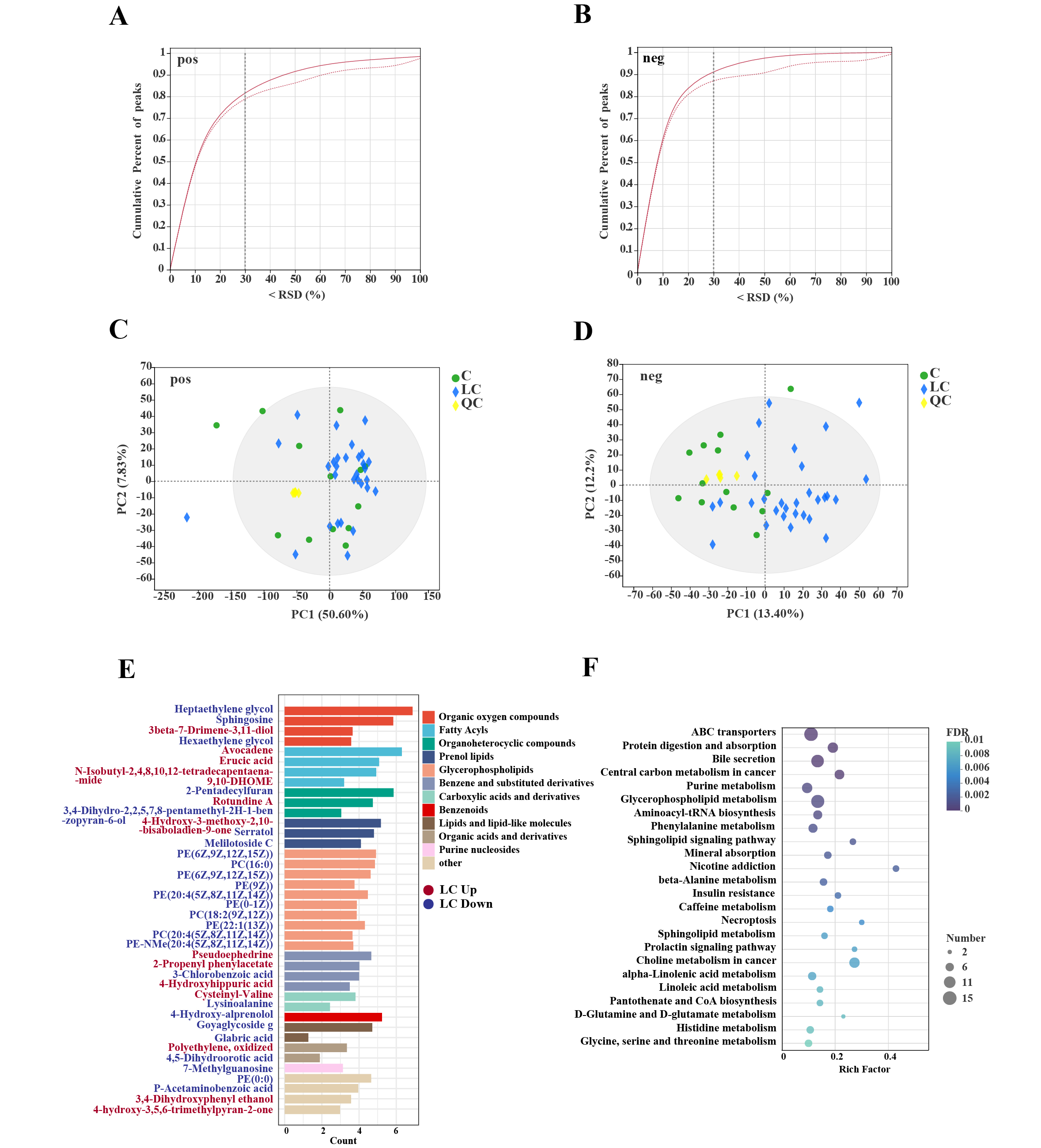

Supplement: Supplementary file 2 [file Image_1.jpeg]

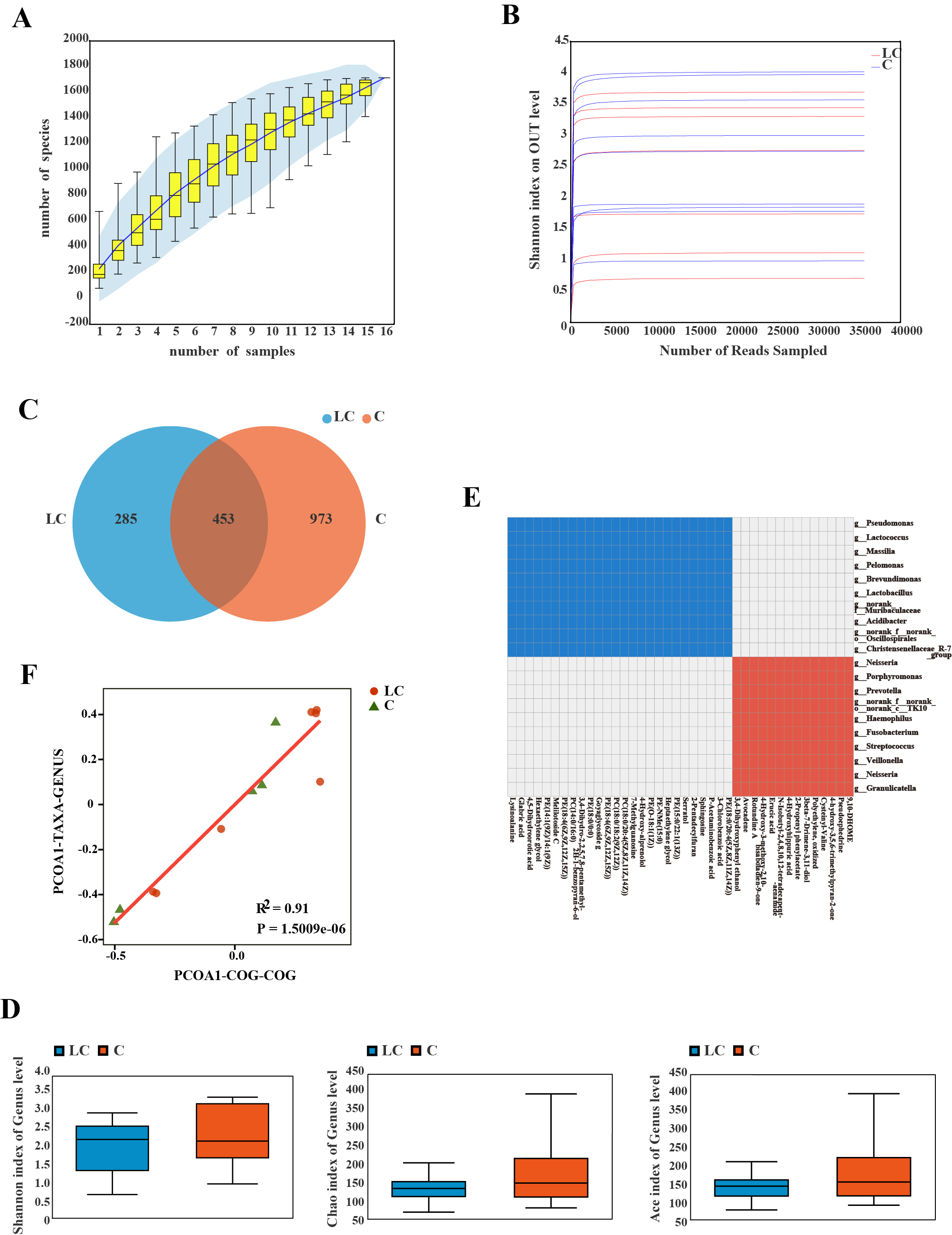

Supplement: Supplementary file 3 [file Image_2.jpeg]

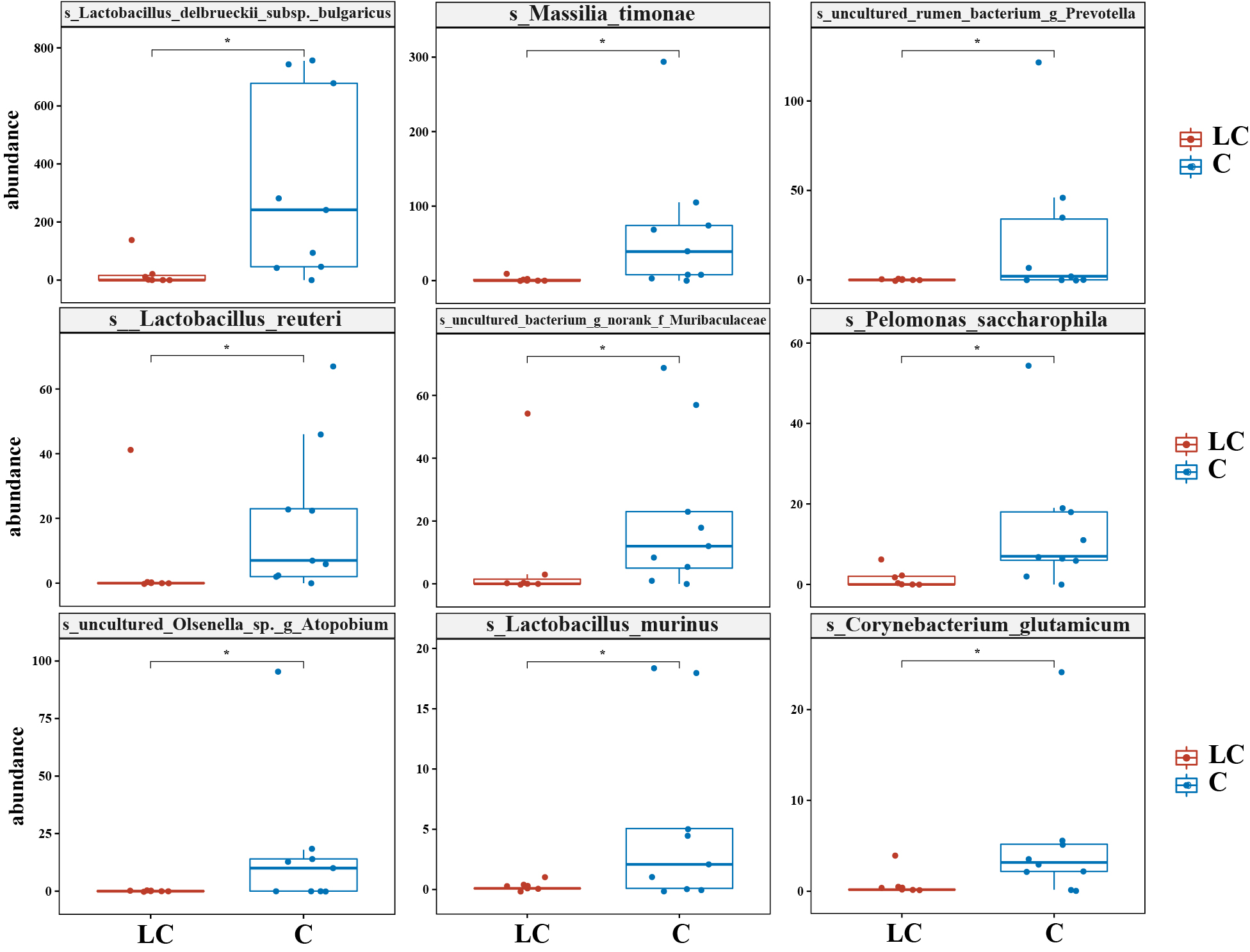

Supplement: Supplementary file 4 [file Image_3.jpeg]
